# Hematopoietic Stem and Immune Cells in Chronic HIV Infection

**DOI:** 10.1155/2015/148064

**Published:** 2015-08-02

**Authors:** Jielin Zhang, Clyde Crumpacker

**Affiliations:** Division of Infectious Diseases, Beth Israel Deaconess Medical Center, Boston, MA 02215, USA

## Abstract

Hematopoietic stem cell (HSC) belongs to multipotent adult somatic stem cells. A single HSC can reconstitute the entire blood system via self-renewal, differentiation into all lineages of blood cells, and replenishment of cells lost due to attrition or disease in a person's lifetime. Although all blood and immune cells derive from HSC, immune cells, specifically immune memory cells, have the properties of HSC on self-renewal and differentiation into lineage effector cells responding to the invading pathogens. Moreover, the interplay between immune memory cell and viral pathogen determines the course of a viral infection. Here, we state our point of view on the role of blood stem and progenitor cell in chronic HIV infection, with a focus on memory CD4 T-cell in the context of HIV/AIDS eradication and cure.

## 1. Introduction 

HIV is a retrovirus, characterized by inserting its genomic DNA into the human genome, followed by the phenotypes of acute, chronic, or latent infection based on the interactions of the viral DNA with a host DNA. Once an HIV DNA is inserted into a host genome, no known immune mechanism so far eliminates the viral DNA from the host genome. Several cellular mechanisms, however, govern the HIV DNA expression after the integration, which regulate retroviral replication and thereby control the disease phenotypes or symptoms of an acute, chronic, or latent infection, including the cellular mechanisms that silence the replication of ancient human endogenous retroviruses (HERVs) [1–10]. Development of highly active antiretroviral therapy (HAART) or combination antiretroviral therapy (cART) has changed the natural course of HIV infection. HAART effectively controls the HIV entry, reverse transcription, integration, package, and even release, except for a direct control of the HIV DNA expression [10].

CD4 T-cell is the target cell of HIV infection. The status of CD4 T-cells, specifically memory CD4 T-cells after HAART, determines the patient anti-HIV immunity, clinical status, and prognosis. HIV DNA expression in memory CD4 T-cells directly governs the activities of an HIV reservoir or the kinetics of the viral reservoir. Moreover, recent studies reveal that memory CD4 T-cells have stem cell properties and preferentially reside and rest in the bone marrow niche [11–20]. Bone marrow, plus stromal cells, and immune cells comprise a niche where hematopoietic stem cells (HSC) reside. Bone marrow is also a niche of hematopoietic progenitor cells (HPC) and now a niche of memory CD4 T-cells and other immune cells [11–20]. Furthermore, the effect of HIV infection on the niche, HSC, HPC, or memory CD4 T-cells has been addressed repeatedly since 1980s. Therefore, we now give this topic a new meaning in line with the functions of the niche and residing cells in a chronic HIV infection after HAART, specifically on their roles in the eradication of HIV and the cure of AIDS.

## 2. Chronic HIV Infection 

Chronic viral infection, by definition, belongs to the category of persistent infection, involves stages of both insidious and productive infection without rapidly killing or even producing excessive damage of the host cells. The other two types of persistent viral infections or persistent virus-host interactions are latent infection and slow infection. The natural course of HIV infection has been identified by using an antiviral drug [21, 22]. Without HAART, HIV develops an acute infection in a host and destroys millions of cells per day, specifically CD4 T-cells, among them, the memory CD4 T-cells [21, 22]. Memory CD4 T-cells have stem cell properties, which supply millions of cells per day via their clonal expansion to fight the invading pathogens. Same as in other viral infections but unlike the others, memory CD4 T-cells dutifully and diligently conduct their clonal expansion and replenish millions of effector cells to fight with the HIV per day. Nonetheless, all these cells turn into fuel to speed up the HIV replication until the memory CD4 T-cell pool is exhausted, by which a chronic infection follows.

With the inception of HAART, the rapid HIV replication in CD4 T-cells is curbed in multiple steps of the viral lifecycle, except on the viral DNA expression [10]. Moreover, the application of HAART pushes the kinetics of HIV infection further into a chronic infection. This not only saves and increases the memory CD4 T-cell pool but also leaves an HIV reservoir based on the feature of a retroviral infection. This viral reservoir is further consolidated when the main stimuli of HIV replication in CD4 T-cells are subdued by HAART, coincidently followed by a deceased clonal expansion of memory CD4 T-cells and a decreased differentiation of effector cells due to the greatly deceased secretions of growth/clonal factors, cytokines, and chemokines, which allow the memory CD4 T-cell to go back to its resting stage [10–20, 23, 24].

It is well known that the essence of adaptive immunity rests on its memory function, manifested mainly by memory CD4 T-cells. In HIV infection, one single memory CD4 T-cell against HIV expands to an anti-HIV clone, supplying millions of effector cells to regulate both cellular and humeral even innate immunities against the HIV infection. In contrast to the natural course of chronic HIV infection in quiescent cells, including memory CD4 T-cells and macrophages, the HAART resulted chronic HIV infection may allow a larger pool of memory CD4 T-cells to harbor the HIV DNA than in a natural HIV chronic infection occurring after the CD4 T-cell exhaustion. The potential pool of memory CD4 T-cells harbor HIV DNA, however, is various and dependent on when, how, and whom HAART is applied to, as well as the genetic derivations of an individual in his/her memory CD4 T-cell clonal formation during the HIV infection. A further elucidating the molecular mechanism of the interplay among memory CD4 T-cell clonal expansion, effector cell differentiation, and HAART application leads to a gateway towards reconstitution of patient anti-HIV immunity, eradication of HIV, and a cure of AIDS.

## 3. Is HSC an HIV Reservoir?

Two points are here crucial for answering this question: what is HSC and what is an HIV reservoir? First, hematopoietic stem cell (HSC) is an adult or tissue stem cell, embodying multipotentiality and self-renewal function. A single HSC can give rise to all lineages of blood and immune cells, reconstituting not only an entire blood system but also the bone marrow niche. Studies in mouse models and bone marrow transplantations in patients have demonstrated this for four decades [25–33]. The methods and techniques that prove HSC multipotentiality have been used to study other types of stem cells, either totipotent or other multipotent stem cells [25–31, 34–43]. One of the methods and technologies is to utilize the DNA marker to define the daughter or progeny cells derived from HSC, which is now used in gene and cell therapies of diseases. Investigators have used different DNA markers or vector transductions of HSC for gene therapy, including but not limited to the treatment of HIV/AIDS. Such studies are represented by the treatment of the Berlin patient and others. The bone marrow transplantations of CCR5-Δ32 stem cells replenish all of the patient CD4 T-cells and bring a cure [27, 28, 33]. The vector transduced HSC has shown lineage differentiation and exhibited anti-HIV effects in its progeny cells detected in the peripheral blood of all experimental subjects [29–32]. Based on the same technologies and principles, on the other hand, if a HIV DNA has been detected in HSC, the HIV DNA is also to be detected in all lineage blood cells or endpoint cells* in vitro* by differentiation experiments, or to be detected in multiple blood and immune cells* in vivo* through the patients. No studies, however, have shown such a result, in contrast to the fact that vector transduced HSC has shown anti-HIV effects in terminal differentiated CD4 T-cells and CCR5 stem cells replenish the entire patient lineage immune cells with a transduced unique DNA marker, CCR5-Δ32 [27–33].

Next, what is an HIV reservoir? Viral reservoir is an anatomical site in which viruses accumulate and persist. HIV reservoir is defined as a cell type or anatomical site where a replication-competent form of the virus accumulates and persists, with more stable kinetic properties than the main pool of actively replicating virus. The same as the other viral reservoirs, HIV reservoir shows the feature of a persistent or chronic infection, specifically under cART or HAART. In other words, an HIV reservoir is a cell type that allows persistence of replication-competent HIV-1 on a timescale of years in patients on optimal antiretroviral therapy [24, 44–47]. Since 1980s, investigators have been studying the relationship of cell types and HIV infection. Whether HSC is an HIV reservoir, however, has only been addressed recently. Unlike the reports on whether or not HIV causes AIDS, the reports on whether or not HSC is a reservoir are regarding two concepts. There appears to be a different standard on assay cells and on definition of an HIV reservoir. It is scientifically important to use one standard on what is HSC and what is progenitor cell, in both experimentation and conclusion. Specifically, studies on progenitor cells are not suitable to reach a conclusion for stem cells. Second, there is a fine definition of the retroviral DNA or a retrovirus and a quiescent host cell and a viral reservoir [23, 24, 44–66]. The key point here, in the context of HIV cure, is clearly that HSC is not a major HIV reservoir based on the present studies [23, 24, 44–66].

Upon reviewing the literature, like other investigators, we have found out that, up to date, there is a lack of experimental data to show that HIV actively replicates in HSC. Second, there is a lack of studies showing that HSC as an HIV reservoir stably and kinetically provides replication competent virus more than the main pool of actively replicating virus. Third, there is a lack of scientific evidence that HIV DNA in HSC is detected in its multilineage endpoint differentiated cells. Certain reports show that bone marrow cells expressing CD34 phenotypic marker contain viral particle or CD133 hematopoietic progenitor cells harbor HIV DNA. The same studies, nonetheless, conclude that bone marrow, not HSC, may serve as a potentially important reservoir of HIV-1, or CD133 hematopoietic progenitor cells (HPC) harbor HIV genomes, in sharp contrast to concluding that HSC is an HIV reservoir [24, 59, 62, 63, 66]. Moreover, other studies have shown that, in addition to HSC, many lineage progenitor cells including but not limited to CD4 progenitor cells reside in bone marrow [11–20]. CD133, also dubbed AC133, may be a good marker for the selection of human placental cord blood stem cells* in vitro. *Nevertheless, the same study shows that the freshly isolated cord blood CD34+AC133+ stem cells are not susceptible to HIV-1 infection and may not be a viral reservoir [66]. Currently, only latently infected resting CD4+ T-cells fit the proposed definition of a reservoir, and more evidence is necessary to demonstrate that other cell types, including hematopoietic stem cells and macrophages, fit this definition. Aiming at an HIV eradication and AIDS cure, we and other investigators have proposed and now insist that the techniques and methodologies for studying of HSC should be utilized to study memory CD4 T-cells and other immune memory cells, peculiarly for translational research and collaborating multidiscipline study [23, 24, 44–66]. Through the well-established models and methods that identify HIV reservoirs, further research is urgently required on potential reservoirs in the central nervous system and the gut-associated lymphoid tissue [44–47].

## 4. Antiviral Therapy and Anti-Inflammatory Drugs on HSPC 

HSC is a self-replenishing source of all blood and immune cells and in the highest hierarchy of blood cell differentiation. Next to HSC are multipotent progenitor cells, named MPP, or sometimes HPC. HSC gives rise to HPC. HPC have limited self-renewal ability and limited multipotentiality for differentiation into different blood cells compared to HSC. HSC and HPC have been dubbed HSPC. This is a more experimental or bench research term than HSC or HPC, respectively, representing a group of experimentally purified blood stem cells (HSC) and progenitor cells (HPC), due to technical and physiological limitations on separation of stem cells from blood cells and the amount of cells that can be used to study and repeat the experiments. Importantly, the HPC in the HSPC means the primitive progenitor cells, which are technically indispensable in separating HSC from blood cells by current bench purification techniques. The HPC in HSPC is defined as and means the preliminary primary progenitor cell and is definitely not the lineage progenitor cell that has no self-renewal ability and is at the much lower position in the hematopoietic differentiation hierarchy than HSC or MPP [10, 24, 34–40, 48].

It is generally recognized that HIV infection affects bone marrow stromal cells as well as the immune cells that reside in the bone marrow niche [11–20, 23, 34–40, 48–58]. Since bone marrow is not only the niche of HSPC, but also the niche of memory CD4 T-cells and other immune cells [11–20, 23, 34–40, 48–58], we will briefly address the restorative effects of antiviral therapy and anti-inflammatory drugs on HSC, memory CD4 T-cells, and the bone marrow niche, elucidating our point of view on how to utilize anti-inflammatory drug, immunotherapy, and multidiscipline approaches towards an HIV eradication and AIDS cure.

HSC has a unique function on resisting the effects of many drugs. In other words, HSC is refractory to drug effects. This unique function of the blood stem cells is based on a protective mechanism that consisted of cell organelles functioning as a pump, which quickly pumps drugs out of HSC or out of niche, whereby no drugs can affect HSC on its function or cell-cycle status easily [37–40]. HAART or anti-inflammatory drugs plus immunotherapies that are effective on cells dwelling in the niche, specifically on memory CD4 T-cells, will benefit the niche and thereby improve the function of HSC in general, but not on HSC directly. Another intrinsic mechanism that protects the genome of HSC from damages caused by stress, radiation, and so forth, is the quiescent status of HSC, through an enhanced prosurvival gene expression and a strong activation of p53-mediated DNA damage repair responses, in which p21^CipWaf1Sdi1^ (p21) plays an important role [37, 38, 41–43].

Antiretroviral therapy, anti-inflammatory therapy, and immunotherapy have shown synergistic effects in the treatment of HIV. Although the detailed mechanisms remain to be elucidated, administration of drugs synergistically will create a new type of combination therapy, which will speed the process in restoring the function of memory CD4 T-cells, HSC, and the niche, and directly contribute to reconstituting or reprogramming the patient immunity. cART remains under development. Many compounds in cART or HAART now have new forms or new members, such as Tivicay (dolutegravir), a new integrase inhibitor, and the new versions of Truvada and tenofovir, which are more effective on certain compartments but with less side effects compared to their old versions. New compound or biologics are now targeting HIV transcription, which was off target for cART but now a target of antiviral drugs specifically of biologics, including but not limited to a class of nucleobase-amino acid conjugates, Tanshinone II A, or cyclin T1 splice variant, targeting or binding to TAR and specifically inhibiting HIV genomic RNA transcription or exportation [67–69]. Antiviral biologics are a new type of drugs in the field, working on either genetic or epigenetic regulations. Biologics have been used in cancer treatment and now applied in anti-HIV therapy, such as anti-PDL1 MPDL3280A, MK-3475, BMS-936559, or broadly neutralizing antibody 3BNC117, reverberating that certain anticancer drugs are used for anti-HIV therapy since the beginning of AIDS epidemic in early 1980s [70–72]. Immunotherapy of AIDS is not new either, which was started before the discovery of HIV. In the beginning, immunotherapy is used to relieve the AIDS symptoms. After the development of HAART, immunotherapy combined with anti-inflammation drugs is applied to treat immune reconstitution inflammatory syndrome (IRIS). Now, immunotherapy combined with anti-inflammation drugs is under study for synergizing with HAART to reconstitute patient immunity.

IRIS is an inflammatory reaction in HIV infected patients after the initiation of antiretroviral therapy, resulting from the restored immunity to specific infectious or noninfectious antigens. The anti-inflammatory drugs have been applied to improve or synergize the effects of cART. Although some anti-inflammatory drugs are now used in immunotherapy in treatment of both AIDS and cancer, the effects of these drugs on immunotherapy, specifically in reconstitution of patient immunity, remain to be further studied. The pivotal issues here are the following: Why does IRIS consist of only the restored immunities to pathogens other than HIV? What is the immune mechanism of a much weaker reaction to HIV combined with a retroviral rebound in the scheduled treatment interruption even after a prolonged HAART? Despite studies showing the synergy of anti-inflammatory drugs with cART, what is the underpinning mechanism? Importantly, what is the role of anti-inflammatory drugs and cART in reconstitution of patient anti-HIV immunity in which cART alone has already failed? We believe these questions are in the minds of many investigators, which cannot be resolved by solely applying the treatments to patients without deciphering the molecular mechanism, regardless of cART or HAART, immunotherapy or gene therapy or cell therapy, unless we perform basic study on the memory CD4 T-cells to pursue the answer, by virtue of the fact that memory CD4 T-cells are not only the target cells of HIV, but also the commander in chief of immune functions. Dysfunction of these cells causes acquired immune deficiency, whereas restoration of these cell functions lays down a foundation of immune reconstitution.

## 5. Antiviral Therapy and Anti-Inflammatory Drugs on Memory CD4 T-Cell Function 

Similar to defining how HIV destroys CD4 T-cells and causes AIDS via using an antiviral drug [21, 22], one can decipher the molecular pathway that restores memory CD4 T-cell function against HIV infection, reconstituting patient anti-HIV immunity* in vivo* via an anti-inflammatory drug or immunotherapy.

**Figure 1:**
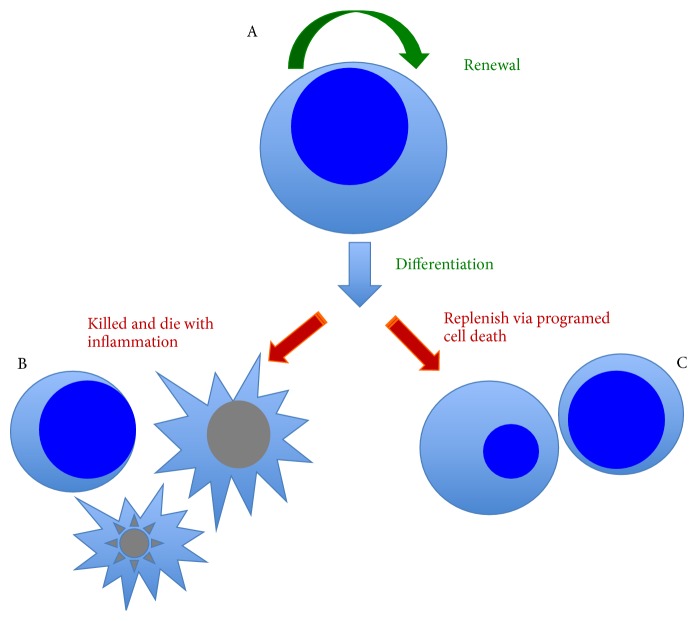
A trinity of cure by CD4 T-cell turnover. P21, cytokine, and immunotherapy can affect all three processes indicated by A, B, and C. Through cell renewal and turnover, one can regulate CD4 T-cell pool size, reprogram T-cell immune memory repertoire, and modulate immune activation and function thereby to reconstitute patient immunity towards an HIV cure.

The hectic clonal expansion of memory CD4 T-cells in HIV infection is a double-edged sword in both AIDS pathology and cure, which not only allows cells to be killed by HIV but also allows cells to die evil via pyroptosis, in sharp contrast to the programmed cell death of immune replenishment via apoptosis (Figure 1) [10, 23, 41–43, 73–79]. How to utilize anti-inflammatory drug or immunotherapy to reprogram memory CD4 T-cell function whereby to reconstitute patient immunity is an imperative task in both the development of AIDS vaccine and cure. Additionally, to elucidate the molecular effects of anti-inflammatory drug and therapy on the function of memory CD4 T-cell, specifically the organelles such as apoptotic body and exosome, will open an avenue to manufacture new immunotherapy drugs and remedies, which are applied not only to the cure of AIDS but also to the other immune diseases such as cancer.

We address this from three aspects briefly. First, we aim to define how to expand the memory CD4 T-cell clone specifically against HIV. Second, we define how big the memory CD4 T-cell repertoire is to execute the HIV specific immunity* in vivo*. Third, we define when and how to apply anti-inflammatory drugs to treat the AIDS symptoms, since, besides AIDS, these symptoms are also observed in patients with cancers and in other immune diseases. Additionally, we believe two issues need to be focused in a near future. One is how to utilize anti-inflammatory drugs to increase memory CD4 T-cell clonal expansion synergizing with HAART. Another is how to use anti-inflammatory drug and immunotherapy to reprogram patient antiretroviral immunity towards a cure of group of immune diseases such as AIDS, cancers, or other immune maladies [10, 24, 42, 48, 80, 81].

## 6. Conclusion 

Aligned with the progress in stem cell research, chronic HIV infection, and the treatment of AIDS, we have proposed our view on whether or not HSC is a major HIV reservoir. We address this based on the established scientific standards, concepts, published data, our own experimental results, and conclusions entailed. Consistent with many other investigators, here we again urge allocating resources to resolve the pressing challenges in HIV/AIDS eradication and cure and to reconstitute host anti-HIV immunity by harnessing well-established techniques and methodologies that have been applied in stem cell research and in viral reservoir study, specifically in defining the reservoirs in an HIV infection.

Furthermore, little is known on the underpinning mechanisms of the currently applied treatments for HIV patients, specifically on immune cell renewal, clonal expansion, and differentiation, including but not limited to cART, immunotherapy, and gene therapy. Resources channeled into such studies will not only unveil the synergy, but also decipher the molecular cellular “synaptic conjunctions” among the varieties of treatment, opening a gateway to reconstitute patient anti-HIV immunity in a sharp contrast to IRIS.

Finally, we propose aiming to resolve the key steps in memory CD4 T-cell clonal expansion. We have focused on applying multidisciplinary expertise and knowledge of stem cell biology, retrovirology, and immunology, from bench to bedside, to elucidate the interplay of clonal expansion and programed cell death in patients with AIDS, with or without HAART. We will address the mechanisms of formation of apoptosis bodies and exosomes in individuals with AIDS, under placebo or anti-inflammatory drugs or immunotherapies. Ultimately, we will bridge the eradication of HIV and cure of AIDS together, fulfilling a cause that reconstitutes or more accurately reprograms patient immunity toward a cure of not only AIDS but also cancer [10, 23, 24, 44–58].
